# Risks and prediction of postoperative hypoparathyroidism due to thyroid surgery

**DOI:** 10.1038/s41598-021-91277-1

**Published:** 2021-06-04

**Authors:** Mustafa Ömer Yazıcıoğlu, Abdurrezzak Yılmaz, Servet Kocaöz, Ruhşen Özçağlayan, Ömer Parlak

**Affiliations:** 1 Department of General Surgery, Breast and Endocrine Surgery Clinics, Ministry of Health Ankara City Hospital, Üniversiteler mah. 1604 cad., No: 9/1, 06800 Çankaya, Ankara Turkey; 2 Department of Internal Medicine Clinic, Ministry of Health Ankara City Hospital, Üniversiteler mah. 1604 cad., No: 9/3, 06800 Çankaya, Ankara Turkey; 3 Department of Internal Medicine Clinic, Balıkesir University Medical Faculty Hospital. Çağış Yerleşkesi, 10145 Bigadiç yolu üzeri 17 km, Balıkesir, Turkey; 4 Department of General Surgery, Yıldırım Beyazıt University School of Medicine, Çankaya, Ankara Turkey

**Keywords:** Biochemistry, Cancer, Biomarkers, Endocrinology, Health care

## Abstract

We aimed to investigate the prevalence of postoperative hypoparathyroidism (PoH), the relevant factors, and predictors of transient or permanent hypoparathyroidism. The files of 352 patients who underwent bilateral total thyroidectomy alone or with central lymph node dissection and/or lateral neck dissection between June 1, 2019, and November 30, 2019, were retrospectively evaluated. Also, calcium and parathyroid hormone levels measured preoperatively and 4–6 h after surgery, follow-up examination results, and time to resolution of transient PoH were recorded. 16.48% (n = 58) of the surgical patients developed transient PoH and 3.98% (n = 14) developed permanent PoH. Length of hospital stay increased in patients who developed PoH (p < 0.001). Transient PoH developed less in patients who underwent parathyroid autotransplantation, while permanent PoH was not detected (p = 0.001). PoH development was not significantly correlated with nodule size as measured by preoperative thyroid ultrasonography (p = 0.944). Patients who had a serum PTH level ≤ 5.95 pmol/L 4–6 h after surgery had a greater risk of developing permanent PoH (OR 134.84, 95% CI 17.25–1053.82). PoH is more common in female gender and is not significantly correlated with nodule size. Parathyroid autotransplantation can prevent the development of PoH.

## Introduction

Hypocalcemia most often develops secondary to hypoparathyroidism, which may result from disrupted integrity of the blood supply to parathyroid glands, or damage due to various reasons or the accidental excision of the parathyroid glands during thyroidectomy^1–3^. Hypocalcemia can also develop due to hungry bone syndrome occurring after Graves' disease surgery^3–4^. Other factors associated with hypoparathyroidism include the experience of the surgeon, retrosternal nodular goiter, postoperative hematoma, and unintended parathyroidectomy during central neck lymph node dissection^4–6^. It is reported that 14–83% of thyroidectomy patients develop transient hypoparathyroidism (< 6 months) while 0.12–11% develop permanent hypoparathyroidism^6,7^. Most patients with transient hypoparathyroidism recover with vitamin D and calcium supplementation for 6 months^8^. Postoperative PTH measurement can diagnose hypoparathyroidism early and prevent unnecessary treatment and allow early discharge^9^. With this study, we aimed to investigate the prevalence of postoperative hypoparathyroidism (PoH), the relevant factors, and predictors of transient or permanent hypoparathyroidism.

## Methods

The files of 352 patients who underwent bilateral total thyroidectomy (BTT) with or without central lymph node dissection (CLND) and/or lateral neck dissection (LND) in Ankara City Hospital between June 1, 2019, and November 30, 2019, were retrospectively evaluated. In our clinic, (83) 24% of patients underwent BTT with minimally invasive thyroid surgery. Patients who underwent thyroid lobectomy and complementary thyroidectomy due to recurrent goiter were excluded from the study. Information regarding age, gender, operation notes, and any postoperative hematoma, seroma, or surgical site infection (SSI) were recorded from examination and follow-up notes. The length of the incision and whether parathyroid autotransplantation was performed or not were recorded from the surgery notes. Hospitalization and discharge dates, pre-and postoperative calcium levels, postoperative 4–6-h parathormone levels, and follow-up calcium and parathyroid hormone values (together with measurement dates) were retrieved from the hospital information management system. PoH is defined as a PTH level < 15 pmol/L measured at 4–6 h postoperatively in our clinic.For patients with transient hypoparathyroidism, the date of the first normal follow-up parathyroid hormone measurement was recorded, and the time to resolution of transient PoH was calculated accordingly. The longest diameter of any thyroid nodules detected in the preoperative ultrasonography was recorded. Informed consent was obtained from all participants who were above 18 years of age.

### Ethical considerations

Before starting the research, the researchers obtained ethical approval from Ankara City Hospital Clinical Research Ethics Committee (Decision No: E-19-217, date: 24.12.2019), and written permission from Ankara City Hospital, where the research was conducted. This study has been performed in accordance with the ethical standards laid down in the 1964 Declaration of Helsinki.

### Statistical analysis

Data were analyzed using the SPSS software package (version 25.0, IBM Corp., Armonk, NY). Data were expressed using descriptive statistics, including numbers, percentages, median, and mean rank. Histopathological data were evaluated using frequencies and percentages. The Kolmogorov–Smirnov test was used to determine whether the data was distributed normally. In the analysis made in terms of patients' ages, it was determined that the data showed normal distribution between the groups (p > 0.05). The student's t-test was used to compare ages between groups. In the analysis of thyroidectomy incision length, nodule diameter (as determined by preoperative USG), and postoperative length of stay, the data were not found to be normally distributed among the groups (p < 0.05). These data were compared using the Mann–Whitney U test. Fisher's Exact and Continuity Correction tests were used for the intergroup comparison of categorical variables. Statistical significance level was accepted as p < 0.05. Risk factors for permanent PoH were identified using logistic regression analysis. For the retrospective model, inclusion criteria for analysis were accepted as 0.01, exclusion criteria being 0.05. Independent variables were included in the analysis by coding. Before regression analysis, receiver operating characteristic (ROC) curve testing was performed to determine the cut-off value for postoperative parathormone levels in patients who developed permanent hypoparathyroidism.

## Results

The study included 352 patients: (79 males and 273 females). 280 thyroidectomy patients did not develop hypocalcemia or hypoparathyroidism, 58 patients (16.48%) developed transient PoH, and 14 (3.98%) developed permanent PoH. 11.4% (n = 9) of male patients and 23.1% (n = 63) of female patients developed hypoparathyroidism (p = 0.035). PoH was significantly more common among female patients. 33.3% (n = 2) of hyperthyroidism (Graves' disease) patients developed hypocalcemia due to the hungry bone syndrome after BTT. Similarly, 40% (n = 5) of patients with preoperative hyperparathyroidism developed hungry bone syndrome. Patients were divided into two groups as PoH (transient or permanent) (group 1) and non-PoH (group 2). Patient ages were distributed normally in both groups. The average age was 47.43 ± 13.04 years for group 1 and 49.15 ± 12.42 years for group 2. The difference between the two groups was not statistically significant (p = 0.299). The mean length of postoperative stay was significantly longer in group 1 (p < 0.001) (Table 1). This finding is attributed to the development of hypocalcemia in patients with PoH. PoH was significantly more common in patients who developed SSI (p = 0.027) (Table 2). The development of transient PoH was higher in patients with papillary thyroid cancer compared to other pathologies (Table 3). In patients with transient PoH, postoperative PTH levels returned to normal in a median of 28 days (range 3–158, IQR 25). Transient hypoparathyroidism developed less in patients who underwent parathyroid autotransplantation, while permanent hypoparathyroidism was not detected (p = 0.001) (Table 4). In patients who underwent parathyroid autotransplantation, PTH levels returned to normal in a shorter time than patients who did not (7 days vs. 32 days) (Table 5). Patients who had a serum PTH level ≤ 5.95 pmol/L 4–6 h after surgery had a 135-fold greater risk of developing permanent PoH (OR: 134.842, 95% CI: 17.254–1053.820) (Table 6). A PTH level of ≤ 5.95 pmol/L 4–6 h after thyroidectomy predicted permanent PoH with a positive predictive value of 100%.

**Table 1:** Comparison of measurements of patients who did and did not develop PoH.

	PoH				No PoH				*P* value
	Median	Min	Max	Mean Rank	Median	Min	Max	Mean Rank	
Time to discharge (days)	4	2	16	252.28	3	1	13	157.01	< 0.001
Incision length (cm)	5.5	2	16	192.40	6	2	15	215.57	0.104
Nodule diameter in preoperative US (mm)	18	1	79.1	210.21	16.25	1	86	209.17	0.944

The statistical significance of the differences between groups was investigated using the Mann–Whitney U test.

*Min*. Minimum, *Max* Maximum, *cm* centimeter, *mm* millimeter.

**Table 2:** Association of some factors with postoperative hypoparathyroidism.

			Hypoparathyroidism		*P* value
			Did not develop PoH, transient or permanent	Developed PoH, transient or permanent	
Gender	Male	n	70	9	0.035*
		%	88.6	11.4	
	Female	n	210	63	
		%	76.9	23.1	
Operation Technique	BTT	n	255	66	0.545**
		%	79.4	20.6	
	BTT + CLND ± LND	n	25	6	
		%	80.6	19.4	
Incision length	≤ 4 cm	n	72	11	0.088*
		%	86.7	13.3	
	> 4 cm	n	208	61	
		%	77.3	22.7	
SSI	No	n	274	66	0.027*
		%	80.6	19.4	
	Yes	n	6	6	
		%	50.0	50.0	
Hematoma or seroma at the incision site	No	n	255	67	0.396**
		%	79.2	20.8	
	Yes	n	25	5	
		%	83.3	16.7	
Pathology result	Benign	n	162	40	0.725*
		%	80.2	19.8	
	Malignant	n	118	32	
		%	78.7	21.3	
Hashimoto's thyroiditis	No	n	241	66	0.141*
		%	78.5	21.5	
	Yes	n	39	6	
		%	86.7	13.3	
PTH	≤ 5.95 pmol/L	n	0	45	< 0.001**
		%	0	100	
	> 5.95 pmol/L	n	280	27	
		%	91.2	8.8	
Total		n	280	72	
		%	79.5	20.5	

*n* Number, *PTH* Parathormone, *BTT* Bilateral total thyroidectomy, *CLND* central lymph node dissection, *LND* lateral neck dissection, *SSI* surgical site infection.

*Continuity Correction test.

**Fisher's Exact test.

**Table 3:** Temporary hypoparathyroidism development according to the histopathological tumor type.

Histopathological type of tumor		Hypoparathyroidism	
		Did not develop PoH, transient or permanent	Developed PoH, transient or permanent
Follicular cancer	n	1	0
	%	100	0
Hurthle cell cancer	n	2	0
	%	100	0
Papillary thyroid cancer	n	108	34
	%	76.1	23.9
Multinodular goiter	n	156	38
	%	80.4	19.6
Diffuse goiter	n	2	0
	%	100	0
Medullary thyroid cancer	n	3	0
	%	100	0
Other thyroid cancers	n	8	0
	%	100	0
Total	n	280	72
	%	79.5	20.5

*n* Number.

**Table 4:** Comparison of the development of temporary hypoparathyroidism in patients with and without parathyroid autotransplantation.

			Hypoparathyroidism		*P* value
			Did not develop PoH, transient or permanent	Developed PoH, transient or permanent	
Parathyroid autotransplantation	No	n	24	63	0.001*
		%	27.6	72.4	
	Yes	n	17	9	
		%	65.4	34.6	
Total		n	41	72	
		%	36.3	63.7	

*n* Number.

*Continuity Correction test used.

**Table 5:** Comparison of postoperative normalization time of PTH levels with and without parathyroid autotransplantation.

	Parathyroid autotransplantation	n	Mean Rank	Min	Median	Max	*P* value
Postoperative normalization of PTH levels (day)	No	49	33.78	7	32	158	< 0.001
	Yes	9	6.22	3	7	14	

*n* Number, Mann–Whitney U test used. *Min*. minimum, *Max.* maximum.

**Table 6:** Enter method for the development of hypoparathyroidism according to postoperative 4–6-h PTH levels.

	B	*P* value	OR	95% C.I. for EXP(B)	
				Lower	Upper
Postoperative 4-h PTH level ≤ 5.95 pg/mL	4.904	**0.000**	**134.842**	17.254	1053.820
Constant	− 5.903	0.000	0.003		

Pseudo (Nagelkerke) *R*^2^ = 0.463, Hosmer–Lemeshow χ2 = 93.718 *p* < 0.001.

Dependent variable: 0 = no permanent hypoparathyroidism, 1 = permanent hypoparathyroidism.

*CI* confidence interval, *OR* odds ratio.

## Discussion

The prevalence of transient PoH appears to be in the same range among our patients as the data reported in the literature^1–3^. It is reported that PoH was more common in patients in which parathyroid glands were intraoperatively identified for in situ preservation^2^. In order to prevent permanent hypoparathyroidism, it is recommended to confirm the presence of the parathyroid glands after thyroidectomy and to autotransplant any unintentionally excised glands^10^. The in situ preservation of the parathyroid glands is of critical importance in preventing permanent PoH after total thyroidectomy^10^. In our clinic, we try to preserve parathyroid glands in situ, and in cases of unintended parathyroidectomy, perform auto-transplantation in the sternocleidomastoid (SCM) muscles.

Hypoparathyroidism is more common with malignancy and in more extensive surgical operations due to delayed surgery^11,12^. In our patients, performing CLND together with BTT did not increase the incidence of PoH. This may be because all patients underwent BTT, and due to our efforts to preserve the parathyroid glands in situ in all cases.

The literature reports a wide range of incidence of transient and permanent PoH and duration of hypocalcemia^11–13^. In our study, total thyroidectomy patients did not receive extra effort to intraoperatively identify the parathyroid glands but special care was taken not to disrupt supply to the glands. In cases of incidental parathyroidectomy, the glands were implanted in the sternocleidomastoid muscle. The mean time to resolution of transient PoH is reported to be 60 days on average^3,13^. In our study, PTH levels of patients who developed PoH returned to normal within 28 days on average. In the context of thyroidectomy, parathyroid glands can regain function even after 1 year provided that the vascular structures and preferably all four glands are preserved^14^.

Karunakaran P et al. reported that 39.4% of patients with Graves' disease developed hypocalcemia due to hungry bone syndrome after BTT^15^. Similarly, the incidence of the hungry bone syndrome was reported to be 14.8–76.3% after parathyroidectomy in patients with primary and secondary hyperparathyroidism^16,17^. Our results are consistent with the literature.

It is reported that post-BTT PoH is more common among females^18,19^. We similarly found that PoH was significantly more common among our female patients. Although it is not clear why the female gender is a risk factor for PoH, it is thought to be due to the low hormonal response of the parathyroid glands^20^.

In patients who develop PoH following BTT, hypocalcemia is corrected by calcium and active vitamin D supplementation, which increases the length of hospital stay by an average of 8–11 days^21,22^. Measuring PTH levels during the postoperative 4–6 h can allow early discharge of patients at low risk of developing hypoparathyroidism and the early treatment of high-risk patients^22^. In our hospital, serum calcium and PTH levels are measured 4–6 h after thyroidectomy as per routine protocol, and thus, our patients were discharged early. As would be expected, the length of hospital stay is increased in patients who develop hypocalcemia.

The literature reports that patients with thyroid nodules > 26 mm are under greater risk of developing malignancy^23,24^. However, to the best of our knowledge, there are no studies investigating the correlation between nodule diameter and PoH. In our study, the diameter of the largest nodule was evaluated in patients with more than one nodule. In the presence of more than one nodule located side by side, the total diameter of the nodules was evaluated. Our results demonstrate that nodule diameter is not correlated with PoH.

Perigli G et al. reported that PoH was significantly less common among patients who underwent minimally invasive thyroidectomy compared to classical incision^25^. In our study, PoH was less common in minimally invasive thyroidectomy (Incision length < 4 cm), but this finding was not statistically significant.

Bleeding is the most common complication associated with thyroidectomy. Surgery secondary to bleeding may cause SSI, hypoparathyroidism, and recurrent nerve palsy^26^. In our study, PoH was not significantly associated with postoperative hematoma or seroma but was significantly higher in patients who developed surgical site infections. We believe this may be ascribed to the impaired supply to the in situ preserved parathyroid glands due to infection. Further large-scale studies are needed to better understand this issue.

The risk of acute PoH increases in patients with a postoperative PTH level < 15 pmol/L and a PTH level < 10 pmol/L measured 4 h or 1 day after surgery indicates an impaired parathyroid metabolism^27–29^. Galy-Bernadoy C et al. reported that the cut-off value of postoperative 4–6-h PTH level to definitively predict permanent PoH with a positive predictive value of 100% was 7 ng/L. Our results show that the risk of developing permanent PoH is increased 135-fold in patients with a postoperative 4–6-h PTH level of < 5.95 pmol/L. Also, a postoperative 4–6-h PTH level of < 5.95 pmol/L has a positive predictive value of 100% for permanent PoH.

## Conclusion

PoH was more common among females and in patients who developed surgical site infections. PoH was not statistically associated with operation technique, malignancy, CLND, incision length, or preoperatively measured thyroid nodule size. The length of hospital stay was longer for patients who developed hypocalcemia, as would be expected. A postoperative 4–6-h PTH level of < 5.95 pmol/L was found to be an early predictor of permanent PoH.
